# Impact of catheter-to-vein diameter ratio on thrombosis in pediatric central venous catheterization

**DOI:** 10.3389/fped.2025.1631247

**Published:** 2025-08-21

**Authors:** Fevzi Kahveci, Nur Ayça Çelik, Hacer Uçmak, Anar Gurbanov, Mert Kaan Coşkun, Hasan Özen, Şükriye Yılmaz, Merve Havan, Suat Fitöz, Mehmet Ertem, Tanıl Kendirli

**Affiliations:** Division of Pediatric Intensive Care, Department of Pediatrics, Ankara University Faculty of Medicine, Ankara, Türkiye

**Keywords:** thrombosis, catheter vessel diameter ratio, central venous catheter (CVC), pediatrics, PICU (pediatric intensive care unit)

## Abstract

**Objective:**

Catheter-related thrombosis is a common complication of central venous catheter insertion. As the use of central venous catheters increases in pediatric critical care settings, catheter-related thrombosis is becoming more common among patients who typically have multiple risk factors for thromboembolism. We aimed to investigate impact of catheter-to-vein diameter ratio on thrombosis in pediatric central venous catheterization.

**Methods:**

Single-center, prospective study. In our study, thrombosis risk factors and patient-related factors were excluded.

**Results:**

A total of 50 patients were included in our study. Thrombosis was observed in 34% of the patients. When comparing thrombotic and nonthrombotic patients, factors such as a low aPTT value, dialysis catheter use, certain mutations that may cause thrombosis, a high catheter-to-blood vessel diameter ratio (C/VR), and a high catheter area-to-blood vessel area ratio (C/VA) are associated with an increased risk of thrombosis. In backwards logistic regression analysis of thrombosis risk, older age, a decreased catheter area, a high C/VA ratio, and the use of dialysis catheters contributed to an increased risk of thrombosis. Patients with dialysis catheters have a 64.9 times greater risk of thrombosis than do those with central venous catheters. The C/VR, with a cut-off value of 0.197, and the C/VA, with a cut-off value of 0.088, are effective indicators in ROC analysis for thrombosis.

**Conclusion:**

In conclusion, selecting a catheter with a diameter-to-vessel diameter ratio of less than 1:5 in normovolaemic paediatric patients should be considered as a strategy to reduce the risk of catheter-related thrombosis.

## 1 Introduction

Catheter-related thrombosis is a common complication of central venous catheter (CVC) insertion. As the use of central venous catheters increases in pediatric critical care settings, catheter-related thrombosis is becoming more common among patients who typically have multiple risk factors for thromboembolism (1). Catheter-related thrombosis involves not only patient-related risk factors but also specific risk factors associated with the catheter. Any catheter has the potential to cause thrombosis (2). Peripherally inserted central catheters (PICC lines), and catheter entry devices with or without tunnels have different rates of thrombosis risk (3–5).

Another critical factor is the catheter diameter-to-blood vessel diameter ratio. The catheter diameter-to-vein diameter ratio determines whether blood flows through or stagnates in the vein. A catheter with a larger diameter has a greater possibility of thrombosis in similar-sized blood vessels than a smaller-sized catheter does. This is the reason that adult studies recommend that the CVC catheter area should not exceed more than 45% of the vessel area (6, 7). Some adult studies suggest that this rate should be decreased to 33% (7, 8).

Central venous catheter-related thrombosis can be categorised into 3 different classes: fibrin sheath thrombosis surrounding the catheter, thrombotic blockage of the catheter lumen, and superficial or deep vein thrombosis. Central line insertion causes local venous damage at the insertion site. Fibrin accumulation on the thrombogenic catheter surface, smooth muscle growth, and endothelial cell proliferation increase hours after catheter insertion (9). The fibrin sheath starts at the site of endothelial damage and grows through the catheter. Blood flow decreases by 60% around the catheter, which results in increased cell adherence to vein walls (10). Fibrin sheath formation typically involves the development of a fibrinous layer around the indwelling catheter, often resulting in functional impairment such as difficulty with aspiration, while infusion may remain possible. In contrast, catheter-related thrombosis (CRT) denotes the formation of a thrombus within the venous lumen, frequently manifesting with overt clinical signs such as limb or neck swelling, localised pain, erythema, and impaired catheter function. Therapeutic interventions likewise vary: fibrin sheath is typically managed with local instillation of thrombolytic agents such as urokinase or alteplase to restore catheter patency. CRT, on the other hand, necessitates systemic anticoagulation—commonly with low-molecular-weight heparin—for a minimum duration of three months due to its potential for serious complications, including pulmonary embolism and post-thrombotic syndrome. The table summarising these differences was included as Supplementary Table S1.

## 2 Methods

### 2.1 Study population and protocol

**Figure 1:**
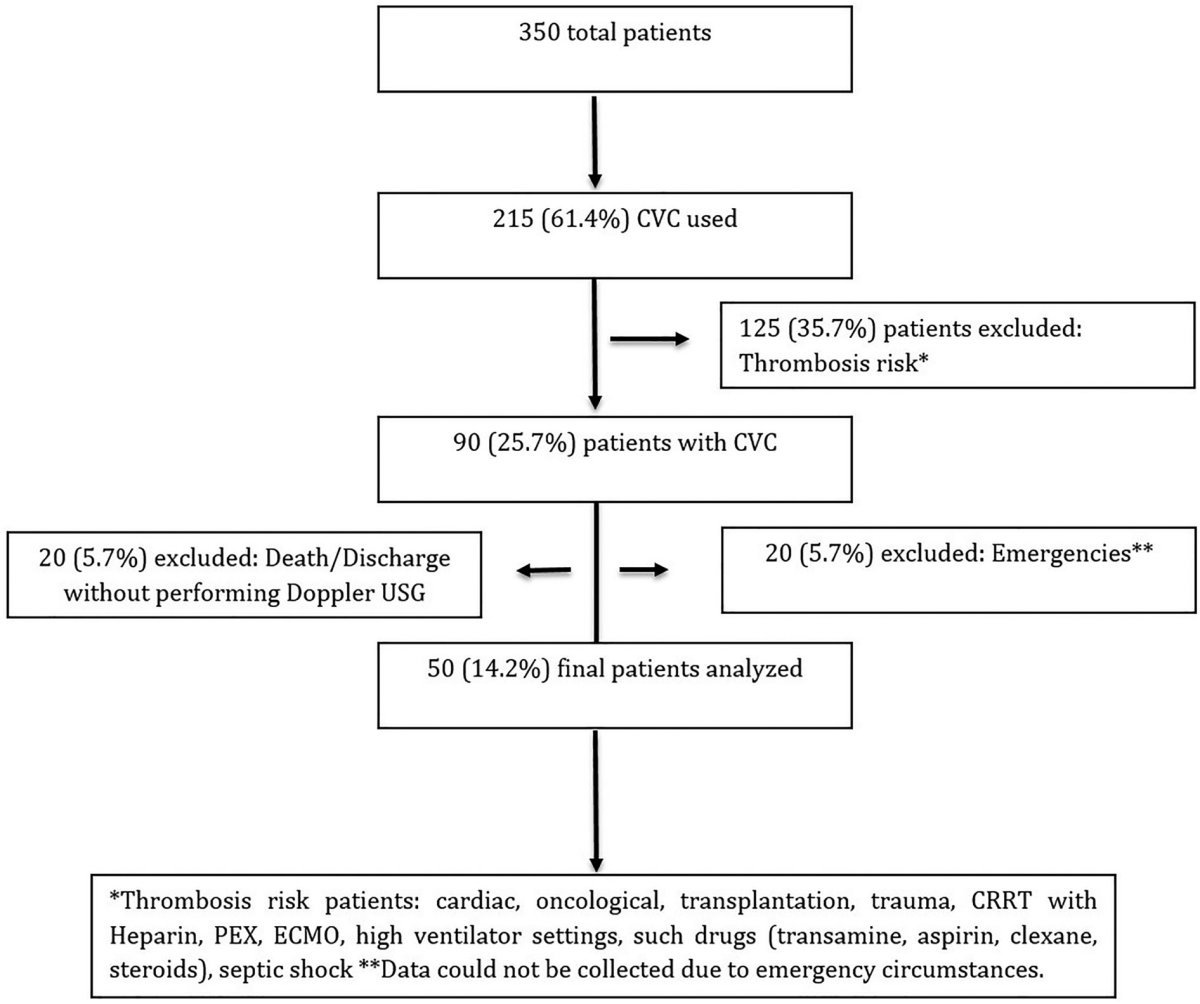
Study flow diagram.

This study is a single-center prospective study conducted between November 2022 and April 2023. Approximately 1,000 catheters are inserted every year in our unit. Fifty patients were included in this study. The flow diagram (in Figure 1) shows how patients were included in the study.

### 2.2 Setting

This study was conducted in a university hospital's pediatric intensive care unit (PICU). In our PICU, intensive care services are provided to patients in internal and surgical departments, and approximately 700 patients are followed annually. Our PICU is a combined unit offering pediatric and cardiac critical care. Written permission was obtained from the local ethics committee of our hospital [ethics committee: İ09-554-22]. Before the procedures, patients were informed about the procedural risks and therapeutic benefits. Written informed consent was obtained from all patients' relatives or legal authorities when necessary. Our study was conducted following the ethical principles of the World Medical Association Declaration of Helsinki.

### 2.3 Study design

#### 2.3.1 Ultrasound examination

In our study, three observants (PICU clinical fellows) took measures before catheter insertion day and night. The radiology department gave observants 2 h of theoretical and practical teaching for standardisation. The measurements were recorded via photographs and then evaluated. The study was started after the consistency of the observants' measurements was observed in 20 patients at the first stage. Measurements were taken while the patient was lying supine, at rest or under sedation. While the patient was in the supine position, the patient's vascular diameter was measured via an ultrasound probe without compression. The FUJIFILM FC1 ultrasound device was used in the study. Linear and cardiac probes were used with specific frequencies. The vascular diameters were obtained from the internal jugular and femoral veins, where CVCs were placed. Catheters placed in the subclavian vein were excluded from the study because they were unsuitable for measurements. Before the measurements, the patient's fluid status was assessed noninvasively. If fluid deficit was suspected, isolated saline, Ringer's lactate, or saline boluses were administered, and if the volume status indicated no fluid deficit, then the vascular diameter was remeasured, and catheterisation proceeded.

While the inferior vena cava collapse index (IVCCI) and the inferior vena cava distensibility index (IVCDI) were measured, measurements were taken under the xiphoid process via a cardiac probe in the appropriate plane at a distance of 0.5 cm from the hepatic vein in M-mode (11). Measurements were taken from standardised anatomical areas for consistency when measuring the vessel diameter and area. Minimum inspiratory and maximum expiratory measurements in M-mode were recorded.

In this study, before catheter placement, in addition to measuring vessel diameter and area, the IVVCI was measured in extubated patients, and the IVCDI was measured in intubated patients to assess whether the patient's volume status was appropriate before catheter placement. Patients were included in the survey only once. Patients who had already had a catheter placed or who had a second catheter inserted in addition to the existing catheter were not included again in the study. During Doppler ultrasound follow-up, if pain, swelling, or edema occurred and the patient became symptomatic, immediate compression ultrasound was performed by a pediatric radiologist. If the patient remained asymptomatic, compression ultrasound was performed after the catheter was removed.

In this study, particular attention was given to differentiating between fibrin sheath formation and CRT in paediatric patients undergoing CVC placement in the intensive care unit. Fibrin sheath was suspected when catheter dysfunction was observed—typically an inability to aspirate despite unimpeded infusion—and confirmed by Doppler ultrasonography showing hyperechoic material enveloping the catheter without intraluminal thrombus or venous non-compressibility. In contrast, CRT was diagnosed based on standard sonographic findings such as non-compressibility of the vein, absence or reduction of flow, and visible echogenic thrombus. All ultrasonographic evaluations were performed by experienced paediatric radiologists, ensuring consistent interpretation and classification. This distinction was made to avoid potential diagnostic overlap and to more accurately assess the mechanical and anatomical factors contributing to CRT.

#### 2.3.2 Follow-up

Age, weight, admission diagnosis, chronic illness, volume status at admission, catheter insertion indication, previous catheter insertion history, number of days of catheter use, size, length, lumen number of the inserted catheter, extracorporeal membrane oxygenation (ECMO), continuous renal replacement therapy (CRRT) or plasma exchange (PEX) with citrate were used, the presence of extremity circulatory disturbance, thrombosis at another site from the catheter site, patient risk factors, and laboratory measurements before catheter removal were evaluated. In patients with detected thrombosis, when thrombosis was identified via Doppler ultrasound, thrombophilia genetic tests were performed [the most commonly identified mutations in the Turkish population include the factor V Leiden mutation, prothrombin 20210A mutation, and methylenetetrahydrofolate reductase (MTHFR) mutation].Central venous catheter (CVC)-related thrombosis may present with various inspection findings depending on catheter location and thrombus extent. Common signs include unilateral limb swelling and asymmetry, particularly with upper extremity or internal jugular vein placement. Involvement of proximal veins or the superior vena cava may lead to facial or neck edema and dilated superficial veins over the chest or shoulder. Local erythema, skin tightness, and increased vascular markings near the catheter site may indicate local venous congestion or inflammation. In femoral catheterization, unilateral leg edema or inguinal ‘’fullness may be observed. While these findings are non-specific, they should prompt suspicion for catheter-associated thrombosis, especially when accompanied by catheter malfunction or limb discomfort. Diagnostic confirmation requires Doppler ultrasonography.

#### 2.3.3 Inclusion and exclusion criteria

Patients were eligible for inclusion if they were receiving any form of respiratory support, regardless of their volume status. Both intubated and extubated patients were considered. In extubated patients, the IVVCI was assessed, while in intubated patients, the IVVDI was measured. Each patient was included in the study only once. Only CVCs that had been in place for more than 24 h were considered eligible for evaluation. In cases where multiple catheters were present during the same hospital admission, only the first inserted catheter was included in the analysis. During Doppler ultrasonography, all catheter exit sites were systematically evaluated. Patients meeting any of the following criteria were excluded from the study: severe tricuspid regurgitation, right heart failure, massive pleural effusion, cardiac tamponade, mechanical ventilation with a positive end-expiratory pressure (PEEP) greater than 8 cm H₂O, congenital thoracic or spinal cord malformations, history of intra-abdominal surgery, presence of intra-abdominal hypertension, history of organ transplantation, or diagnosis of an oncological disease and patients who were receiving heparin, enoxaparin (Clexane), aspirin, or total parenteral nutrition were also recorded.

#### 2.3.4 Statistical analysis

The analyses were performed with SPSS (Statistical Package for the Social Sciences; SPSS Inc., Chicago, IL) version 22. Descriptive data in the study are presented as frequencies and percentages (n, %) for categorical variables and as median interquartile ranges (25–75 percentile values) for continuous variables. The categorical variables were compared between groups via the chi-square test (Pearson chi-square test). The compatibility of the distribution of continuous variables was assessed via the Kolmogorov‒Smirnov test. The Mann‒Whitney *U*-test was used to compare two groups, whereas the Kruskal‒Wallis test was used to compare more than two groups. Receiver operating characteristic (ROC) curves were drawn to measure the diameter and area in relation to the presence of thrombosis. Backwards logistic regression analysis was performed to calculate the risk of thrombosis. A significance level of *p* < 0.05 was considered statistically significant.

## 3 Results

**Figure 2:**
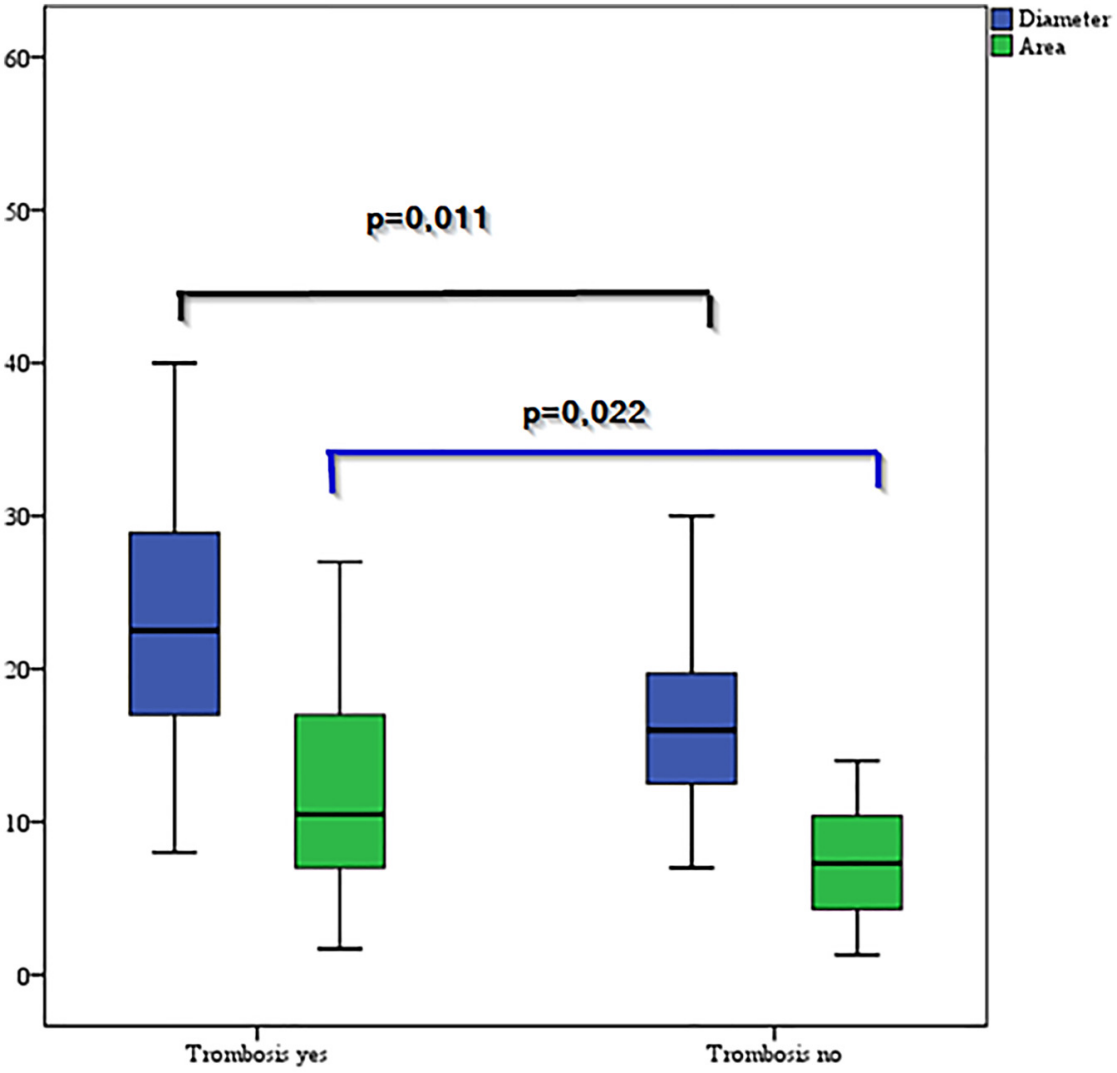
Comparison of the diameter and area according to the presence of thrombosis.

A total of 50 patients were included in the study, with 19 girls (38%) and 31 boys (62%), and thrombosis was observed in 34% of the patients. Thrombosis was detected in 47.4% of the girls and 25.8% of the boys, with no significant difference (*p* = 0.11). The median age of the patients was 45.0 (range 7.0–138.0) months, and there was no significant difference in age according to the presence of thrombosis (*p* = 0.15). In patients with CVCs, the thrombosis rate was significantly lower (27.9%) than that in patients with dialysis catheters (71.4%) (*p* = 0.03). The aPTT value was significantly lower in patients with thrombosis than in those without thrombosis (*p* = 0.01). In patients with mutations, the thrombosis rate was considerably higher (71.4%) than that in patients without mutations (27.9%) (*p* = 0.03). In patients with thrombosis, the catheter radius-to-vessel radius ratio (C/VR) (*p* = 0.01) and the catheter area-to-vessel area ratio (C/VA) (*p* = 0.02) were significantly greater than those in patients without thrombosis (Table 1, Figure 2).

**Table 1:** Comparison of All parameters based on the presence of thrombosis.

Variable		Thrombosis, yes (*n* = 17)		Thrombosis, no (*n* = 33)		Total (*n* = 50)		p*
		n	%	n	%	n	%	
Gender	Female	9	47,4	10	52,6	19	38,0	0,11
	Male	8	25,8	23	74,2	31	62,0	
Age (months)		84,0 (15,0–180,0)		18,0 (7,0–108,0)		45,0 (7,0–138,0)		0,15**
Height (cm)		105,0 (73,0–155,0)		80,0 (62,0–130,0)		94,5 (62,0–140,0)		0,20**
Weight (kg)		25,0 (10,0–50,0)		12,0 (7,0–25,0)		15,0 (7,0–40,0)		0,09**
BMI (kg/m^2^)		18,8 (16,6–20,9)		18,3 (14,7–20,0)		18,4 (15,0–20,9)		0,44**
Diagnosis	Trauma	1	20,0	4	80,0	5	10,0	0,14
	Neurologic	5	45,5	6	54,5	11	22,0	
	Respiratory	1	9,1	10	90,9	11	22,0	
	Sepsis	2	25,0	6	75,0	8	16,0	
	Other	8	53,3	7	46,7	15	30,0	
Chronic Illness	Yes	11	36,7	19	63,3	30	60,0	0,62
	No	6	30,0	14	70,0	20	40,0	
Laboratory								
aPTT (seconds)		29,4 (27,4–30,3)		32,4 (27,9–39,0)		30,3 (27,6–35,4)		0,01**
INR (ratio)		1,1 (1,1–1,3)		1,2 (1,1–1,3)		1,2 (1,1–1,3)		0,38**
Hemoglobin (g/dl)		10,3 (9,1–12,7)		10,6 (9,7–11,6)		10,5 (9,3–11,8)		0,96**
Platelets (×10⁹/L)		275 (79–346)		250 (148–406)		252.5 (124–397)		0,51**
CRP (mg/dl)		28,4 (4,8–67,5)		21,2 (2,9–72,4)		21,7 (3,1–72,4)		0,72**
Femoral catheter	Yes	2	66,7	1	33,3	3	6,0	0,26
	No	15	31,9	32	68,1	47	94,0	
Left jugular catheter	Yes	3	37,5	5	62,5	8	16,0	0,82
	No	14	33,3	28	66,7	42	84,0	
Catheter type	CVC	12	27,9	31	72,1	43	86,0	**0,03**
	Dialysis Catheter	5	71,4	2	28,6	7	14,0	
Days of Catheter Used		10,0 (8,0–20,0)		11,0 (7,0–22,0)		10,5 (7,0–21,0)		0,84**
Number of catheter insertion attempts		1,0 (1,0–2,0)		1,0 (1,0–2,0)		1,0 (1,0–2,0)		0,89**
Length of catheter (cm)		13,0 (10,0–13,0)		8,0 (8,0–13,0)		10,5 (8,0–13,0)		0,05**
Lumen number of catheter		2,0 (2,0–3,0)		3,0 (2,0–3,0)		3,0 (2,0–3,0)		0,36**
Size of catheter (French)		7,0 (5,0–7,0)		5,0 (5,0–7,0)		5,3 (5,0–7,0)		0,06**
External catheter cross-sectional area		4,1 (2,2–4,1)		2,2 (2,2–4,1)		2,7 (2,2–4,1)		0,05**
Mutation factor	Yes	5	71,4	2	28,6	7	14,0	**0,03**
	No	12	27,9	31	72,1	43	86,0	
C/V diameter		22,5 (17,0–28,9)		16,0 (12,5–19,7)		17,0 (13,0–22,9)		0,01**
C/V area		10,5 (7,0–17,0)		7,3 (4,3–10,4)		8,1 (5,0–12,8)		0,02**

C/V, Catheter/Vessel.

*Chi-square test, **Mann–Whitney *U*-test applied.

**Figure 3:**
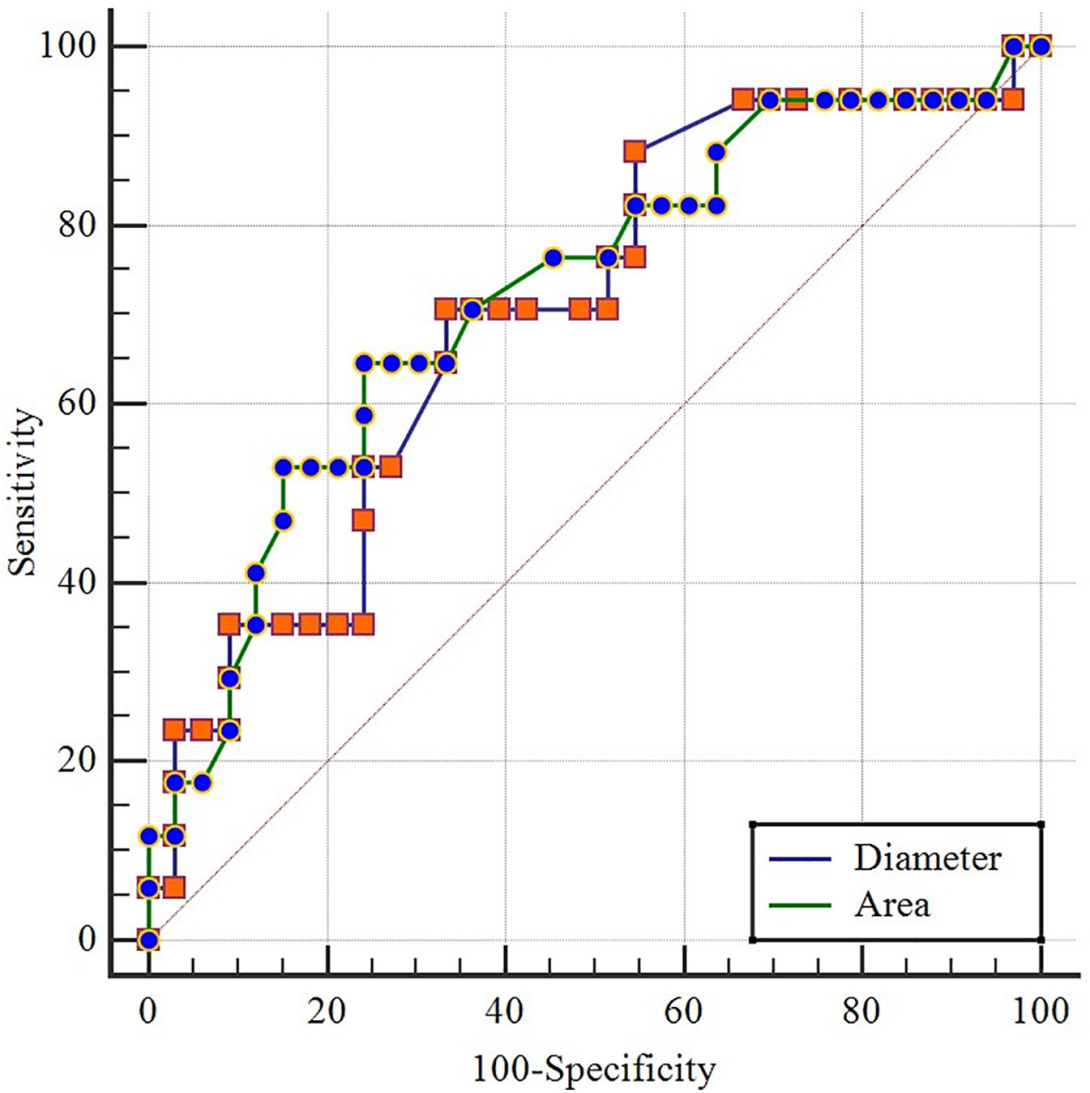
ROC curves of vessel diameter and cross-sectional area for the detection of thrombosis.

The ability of the diameter and area to predict thrombosis was investigated via ROC curve analysis, and the cut-off values were determined. For the C/VR, a cut-off value of 0.197 showed 64.7% sensitivity and 75.8% specificity, indicating that it is a good predictor. For C/VA, a cut-off value of 0.088 had 70.6% sensitivity and 66.7% specificity, indicating that it is a good predictor (Table 2, Figure 3).

**Table 2:** Sensitivity and specificity of diameter and area in determining the presence of thrombosis.

Ratios	Area	p	%95 trust margin		Sensitivity	Specicity	PPD	NPD
			Lower limit	Upper limit				
C/VR > 0.197	0,721	**0,00**	0,576	0,839	64,7	75,8	57,9	80,6
C/VA > 0.088	0,699	**0,01**	0,553	0,820	70,6	66,7	52,2	81,5

There was a significant difference in age between the thrombosis groups, which originated from the fibrin group, and the other two groups, as the fibrin group was younger (*p* = 0.04). Significant differences were observed between the thrombosis groups in terms of catheter length (*p* = 0.04), C/VR (*p* = 0.01), and C/VA(*p* = 0.01), with these differences originating solely from the difference between the thrombosis group and the fibrin group, where the thrombosis group had higher values. Among patients with mutations, thrombosis was observed in 71.4% and fibrin in 28.6%, whereas among patients without mutations, thrombosis was absent in 62.8%, present in 27.9%, and fibrin was observed in 9.3%, with a significant difference between the two groups (*p* = 0.00) (Table 3).

**Table 3:** Comparison of all parameters according to thrombosis and fibrin sheath status.

Variable		None (*n* = 27)		Thrombosis, yes (*n* = 17)		Fibrin sheath, yes(*n* = 6)		p*
		Mean (IQR)		Mean (IQR)		Mean (IQR)		
Age (months)		24,0 (9,0–138,0)^a^		84,0 (15,0–180,0)^a^		4,8 (4,0–10,0)^b^		**0,04**
Height (cm)		85,0 (65,0–140,0)		105,0 (73,0–155,0)		60,5 (55,0–65,0)		0,07
Weight (kg)		14,5 (7,0–38,0)		25,0 (10,0–50,0)		8,6 (5,5–10,0)		0,10
Chronic ilness	Yes	16	53,3	11	36,7	3	10,0	0,85**
	No	11	55,0	6	30,0	3	15,0	
Femoral catheter	Yes	1	33,3	2	66,7	0	,0	0,69**
	No	26	55,3	15	31,9	6	12,8	
Left jugular catheter	Yes	5	62,5	3	37,5	0	,0	0,74**
	No	22	52,4	14	33,3	6	14,3	
Catheter type	CVC	25	58,1	12	27,9	6	14,0	0,11**
	Dialysis catheter	2	28,6	5	71,4	0	,0	
Simultaneous catheter	Yes	4	80,0	1	20,0	0	,0	0,81**
	No	23	51,1	16	35,6	6	13,3	
Catheter insertion day		2,0 (1,0–6,0)		3,0 (2,0–17,0)		5,0 (1,0–34,0)		0,37
Total duration of catheter (days)		12,0 (7,0–26,0)		10,0 (8,0–20,0)		7,5 (6,0–11,0)		0,35
Number of catheter insertion attempts		1,0 (1,0–1,0)		1,0 (1,0–2,0)		2,0 (1,0–3,0)		0,06
Length of Catheter (cm)		10,0 (8,0–13,0)^a^^,^^b^		13,0 (10,0–13,0)^a^		8,0 (8,0–8,0)^b^		**0,04**
Lumen number of catheter		3,0 (2,0–3,0)		2,0 (2,0–3,0)		3,0 (2,0–3,0)		0,62
Size of Catheter (French)		5,0 (5,0–7,0)		7,0 (5,0–7,0)		5,0 (4,0–5,0)		0,12
External catheter cross-sectional area		2,2 (2,2–4,1)		4,1 (2,2–4,1)		2,2 (1,3–2,2)		0,10
Mutation factor	Yes	0	,0	5	71,4	2	28,6	0,00**
	No	27	62,8	12	27,9	4	9,3	
C/V diameter		17,0 (13,0–21,0)^a^^,^^b^		22,5 (17,0–28,9)^a^		10,4 (9,2–13,0)^b^		**0,01**
C/V area		8,0 (5,0–12,5)^a^^,^^b^		10,5 (7,0–17,0)^a^		3,1 (2,8–4,3)^b^		**0,01**

*Kruskal–Wallis test and **Chi-square test were applied. Groups marked with ^a,b^indicate where the difference originates.

In the backwards logistic regression analysis for calculating thrombosis risk, older age, a lower external catheter cross-sectional area, and a higher C/VA were identified as risk factors for thrombosis (Table 4). The model included sex, age, aPTT value, catheter type, catheter length, external catheter cross-sectional area, whether a mutation increased the risk of thrombosis, and C/VR and C/VA.

**Table 4:** Logistic regression analysis of the presence of thrombosis (50 patients).

Variable	B	S.E.	p	OR	95% C.I. for OR	
					Lower	Upper
Age	,025	,011	**0,02**	1,025	1,003	1,048
APTT	-,194	,108	0,07	,824	,667	1,018
Catheter type (reference = CVC)	4,058	2,161	0,06	57,852	,838	3,995,564
External catheter cross-sectional area	-,995	,485	**0,04**	,370	,143	,955
Mutation factor (reference = no)	2,165	1,245	0,08	8,715	,760	99,990
Catheter vessel area rate	,177	,076	**0,02**	1,194	1,028	1,387

In the logistic regression analysis for thrombosis risk, older age, a lower external catheter cross-sectional area, and a higher C/VA were found to increase the risk of thrombosis. A dialysis catheter instead of a CVC increased the risk of thrombosis by 64.955 times (Table 5). The model included sex, age, aPTT value, catheter type, catheter length, catheter outside area, C/VR, and C/VA.

**Table 5:** Logistic regression analysis of the presence of thrombosis (45 patients).

Variable	B	S.E.	p	OR	95% C.I. for OR	
					Lower	Upper
Age	,020	,010	**0,04**	1,020	1,000	1,040
Catheter type (reference = CVC)	4,174	1,959	**0,03**	64,955	1,396	3023,014
External catheter cross-sectional area	-,915	,453	**0,04**	,401	,165	,973
Catheter vessel area rate	,153	,068	**0,02**	1,166	1,020	1,332

## 4 Discussion

In critically ill patients, CVCs or dialysis catheters are required for medical treatment and procedures such as CRRT and PEX. While these procedures benefit patients, they can also lead to increased morbidity and mortality due to their direct and indirect effects. In modern pediatric intensive care, performing procedures under ultrasound guidance helps prevent complications during catheter insertion. It may also reduce complications such as pulmonary embolism, which can develop in the context of thrombosis. In this study, we aimed to investigate how catheter and vessel size, which are well defined in adult intensive care practice, are related to thrombosis in pediatric ICU patients. We hypothesised that instead of selecting standard catheter sizes on the basis solely of the patient's weight and height, considering the patient's volume status would allow the selection of the most appropriate catheter size on the basis of the vessel diameter and area, ultimately reducing catheter-related complications. In our study, the incidence of thrombosis was 34%, with dialysis catheters identified as the most decisive risk factor.

### 4.1 Comparison with prior literature

In a study by Kim and colleagues investigating thrombosis incidence and contributing factors in 80 patients under the age of 6, the incidence of thrombosis was 38.8%. In the logistic regression analysis, the only significant factor was the number of catheter insertion attempts, with more than two attempts statistically associated with thrombosis (12). In a study by Kujur et al. investigating the incidence of central venous catheter-related thrombosis in 100 adult patients, intermittent Doppler ultrasound monitoring of internal jugular venous CVCs revealed a 33% thrombosis rate (13). In our study, the incidence of thrombosis was found to be 34%. Four patients had three attempts at catheter insertion, two of whom had fibrin sheaths, and one had thrombosis. However, the number of insertion attempts was not a significant risk factor for thrombosis in our study. In a review by Jaffray et al. on catheter-related thrombosis, anatomically placed catheters were more likely to be associated with thrombosis than those placed under ultrasound guidance (14). We believe that when experienced operators perform procedures under ultrasound guidance and fewer attempts are made, there is no significant difference in thrombosis risk.

In their article on catheter-related thrombosis, Wall et al. associated patient-related factors such as conditions that increase hypercoagulability (sepsis, malignancy, and renal failure), hereditary thrombophilia, and certain medications with an increased risk of thrombosis. They also reported that increased catheter lumen count, catheter length, subclavian catheters, femoral and left jugular catheters, and more insertion attempts contributed to increased catheter-related thrombosis (1). In our study, patients with conditions that increase hypercoagulability, such as sepsis, malignancy, renal failure, thrombophilic disorders, and certain medications, were excluded, and the effects of mechanical factors on thrombosis and fibrin sheath formation were explored. In contrast to the literature, no associations were found between the number of catheter lumens, femoral or left jugular catheter placement, or the number of insertion attempts and catheter-related thrombosis in our study.

In a case‒control study by Tripodi et al. involving 605 patients with venous thromboembolism and 1,290 control subjects, a shortened aPTT was found to be independently associated with venous thromboembolism (15). However, in a study by Shervinrad et al. examining 239 patients with short PTs and aPTT, no venous thromboembolism was observed. They concluded that short aPTT cannot be used as a predictor, as it may result from increased levels of factors II, VIII, IX, XI, fibrinogen, and factor VII (16). In a study by Senthil et al., 40 patients with central venous catheters who developed thrombosis were compared with a control group, and a shorter aPTT was found in the thrombosis group (17). Consistent with the studies by Tripodi and Senthil, our study revealed significantly shorter aPTT in the thrombosis group. Although Shervinrad and colleagues argued that a short aPTT could not be a predictor, we believe that in selected and at-risk patient groups, a short aPTT should be considered a warning sign for thrombosis.

In a study by Cirstoveanu et al. in Romania, thrombophilia panels were performed on 40 neonates, and heterozygous MTHFR mutations were found in 30 patients, whereas homozygous MTHFR mutations were found in 10 patients. They recommended screening for MTHFR mutations in all ICU patients to prevent thrombosis (18). Genetic factors account for approximately 60% of deep vein thrombosis (DVT) cases, with notable thrombophilia defects, including mutations in the MTHFR gene, the factor V Leiden (G1691A) mutation, and the prothrombin G20210A mutation (19). Park et al. examined 146 venous and arterial thrombosis patients and studied homocysteine and MTHFR C677T polymorphisms. They reported that plasma homocysteine concentrations were significantly higher in TT genotypes than in CC genotypes (20). In our study, 2 of the 7 patients with genetic mutations identified by the thrombophilia panel were in the group with fibrin sheath detection and 5 in the group with thrombosis. The thrombophilia panel was not applied to all patients. Mutations were studied in 23 patients with fibrin sheath and thrombosis detected by Doppler USG. The mutations identified included Factor V Leiden and Prothrombin 20210 heterozygous mutations, a heterozygous MTHFR mutation, a combination of MTHFR and Factor V Leiden mutations, a homozygous MTHFR mutation, and a homozygous Prothrombin 20210 mutation. In addition, two patients with genetic mutations were found to have a fibrin sheath. Genetic mutations were identified in 29% of patients with thrombosis in our study.

### 4.2 Pathophysiology

Thrombosis pathogenesis is multifactorial. Traditionally, pathogenic factors are defined in Virchow's classical triad: endothelial injury, reduced venous flow, and hypercoagulability (21). The first two factors are more technical, whereas the last factor is less modifiable and depends on the patient's characteristics and comorbidities (e.g., malignancy, thrombophilic states). A key point here is distinguishing thrombosis from fibrin sheath formation. Thrombosis involves tissue related to endothelial injury, whereas a fibrin sheath is a catheter-related structure formed due to the presence of a foreign object in the vessel. Fibrin sheath formation starts early after catheter placement and is completed within two weeks (22). Thrombosis, on the other hand, begins 24 h after catheter insertion when fibronectin released from liver tissue accumulates (23). Both processes gradually form connective tissue around the catheter. While fibrin sheath formation is a foreign body reaction to the catheter, thrombosis involves endothelial injury and continues through tissue factor-mediated vascular repair. Despite having different pathophysiological processes, their relationship remains unclear (24). As the fibrin sheath extends distally, it covers the catheter tip, potentially causing dysfunction by acting as a valve, allowing injections but preventing blood flow (25). In our study, thrombosis was observed in 17 (34%) patients, and a fibrin sheath was observed in 7 (14%) patients. Fibrin sheaths are more common in younger children, and the risk of thrombosis increases with age. Compared with those in the fibrin sheath group, the catheter length, C/VR, C/VA, and mutation frequency in the thrombosis group were significantly different. Contrary to expectations, there was no significant relationship between the number of attempts and the formation of fibrin sheaths. The presence of fibrin sheaths in younger children may be related to the mechanical effects of the catheter needle, dilator, and catheter on small vessels in this age group.

### 4.3 Study limitations

Owing to the nature of the study, measurements that should have been taken before catheter insertion could not be obtained for many of the catheters inserted in emergencies or during night shifts, even though the patient profile was suitable for the study. These patients were excluded from the study due to the inability to perform initial ultrasonographic evaluations.Patients who were discharged, transferred between units, or died during the night and weekend shifts were excluded from the study because Doppler measurements could not be performed during those times. The patients who passed away, were transferred to the ward, or were discharged without undergoing Doppler ultrasonography were excluded from the study, as thrombosis screening could not be performed. Since genetic mutations and factor levels were observed in cases of thrombosis, factor levels could not be monitored prior to catheter insertion. Another limiting factor was the failure to measure the patients' homocysteine levels. Additionally, we did not use IVCCI and IVCDI measurements because of variability across different age groups, the lack of clear cut-off values for the pediatric age group, and our study's relatively small sample size for this specific analysis. We also consider it a limitation that the study did not assess the anatomical location of the catheter tip (whether it was positioned against the vessel wall or in a cardiac region).

## 5 Conclusion

Our study investigating central venous catheter-associated thrombosis revealed that the C/VR and C/VA ratios were greater in patients with thrombosis. ROC analysis revealed that a C/VR cut-off of 0.197 and a C/VA cut-off of 0.088 were good predictors for thrombosis. Although the sensitivity and specificity values for the C/VR and C/VA thresholds demonstrate statistically acceptable predictive performance, their clinical utility should also be considered. In real-time practice, these thresholds may serve as anatomical indicators that can guide catheter size or placement decisions, potentially reducing thrombosis risk. Consistent with the literature, patients with thrombosis had lower aPTT values. In the backwards logistic regression analysis performed to calculate thrombosis risk, older age, a smaller catheter area ratio, a higher C/VA, and the presence of a dialysis catheter were found to be risk factors for thrombosis. In conclusion, selecting a catheter with a diameter-to-vessel diameter ratio of less than 1:5 in normovolaemic paediatric patients should be considered as a strategy to reduce the risk of catheter-related thrombosis.

## Data Availability

The raw data supporting the conclusions of this article will be made available by the authors, without undue reservation.

## Supplementary material

Supplementary Table S1 Comparison Between Fibrin Sheath and Catheter-Related Thrombosis.
