# Supplementary material for: Impact of catheter-to-vein diameter ratio on thrombosis in pediatric central venous catheterization
Source: Front Pediatr. 2025 Aug 21;13:1631247. doi: 10.3389/fped.2025.1631247 (PMC12408594; doi:10.3389/fped.2025.1631247)
Supplement: Supplementary Table S1 — Comparison Between Fibrin Sheath and Catheter-Related Thrombosis. [file Table1.pdf]

Supplementary Table 1: Comparison Between Fibrin Sheath and Catheter-Related Thrombosis

Feature	Fibrin Sheath	Catheter-Related Thrombosis
Pathophysiology	Formation of a fibrinous layer around the catheter	Thrombus formation within the vessel lumen
Clinical Presentation	Typically only aspiration difficulty	Aspiration issues plus visible swelling, pain, erythema
Imaging	Difficult to detect; may require linogram	Detected reliably with Doppler ultrasound
Treatment	Local thrombolytics (e.g., urokinase, alteplase)	Systemic anticoagulation for at least 3 months
Clinical Significance	Primarily functional impairment	Risk of thromboembolism, associated morbidity
